# Formulation and In Vitro Assessment of Polymeric pH-Responsive Nanogels of Chitosan for Sustained Delivery of Madecassoside

**DOI:** 10.1021/acsomega.4c00461

**Published:** 2024-04-16

**Authors:** Muhammad Suhail, I-Hui Chiu, Arif Ullah, Arshad Khan, Hamid Ullah, Noorah Saleh Al-Sowayan, Pao-Chu Wu

**Affiliations:** † School of Pharmacy, Kaohsiung Medical University, 100 Shih-Chuan first Road, Kaohsiung 80708, Taiwan; ‡ Institute of Biomedical Materials, School of Materials Science and Engineering, Zhejiang Sci-Tech University, Hangzhou 310018, China; § Department of Biotechnology, University of Science and Technology Bannu, Bannu 28100, Pakistan; ∥ Department of Pharmaceutics, Faculty of Pharmacy, The Islamia University of Bahawalpur, Khawaja Fareed Campus (Railway Road), Bahawalpur 63100, Pakistan; ⊥ Department of Biology, College of Science, Qassim University, Buraydah, 52571 Saudi Arabia; # Department of Medical Research, Kaohsiung Medical University Hospital, Kaohsiung 80708, Taiwan; ∇ Drug Development and Value Creation Research Center, Kaohsiung Medical University, Kaohsiung 80708, Taiwan

## Abstract

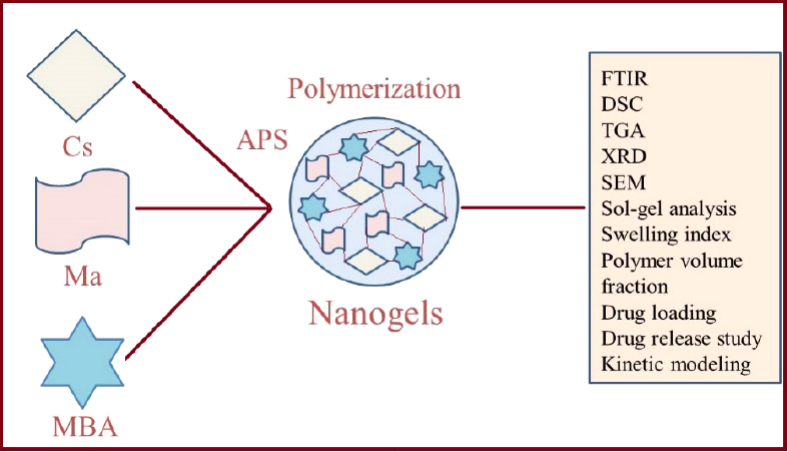

Madecassoside, a triterpenoid saponin compound mainly isolated from the gotu kola herb (*Centella asiatica*), shows an extensive range of biological activities, including antiapoptotic, antioxidant, anti-inflammatory, moisturizing, neuroprotective, and wound healing effects. It has been highly used in the management of eczema, skin wounds, and other diseases. Due to poor oral bioavailability, membrane permeability, and intestinal absorption, the clinical application of the madecassoside is limited. Hence, a drug carrier system is needed that not only sustains the release of the madecassoside but also overcomes the drawbacks associated with its administration. Therefore, the authors prepared novel pH-responsive chitosan-based nanogels for the sustained release of madecassoside. Free radical polymerization technique was used for cross-linking of polymer chitosan and monomer methacrylic acid in the presence of cross-linker N′,N′-methylene bis(acrylamide). The decrease in polymer crystallinity after polymerization and development of nanogels was demonstrated by XRD and FTIR analysis. The effects of nanogel contents on polymer volume, sol–gel analysis, swelling, drug loading, and release were investigated. Results indicated that high swelling and maximum release of the drug occurred at pH 7.4 compared to pH 1.2 and 4.6, indicating the excellent pH-sensitive nature of the engineered nanogels. High swelling and drug release were perceived with the integration of a high quantity of chitosan, while a decline was observed with the high integration of N′,N′-methylene bis(acrylamide) and methacrylic acid contents. The same effects of nanogel contents were shown for drug loading too. Sol fraction was reduced, while gel fraction was enhanced by increasing the chitosan load, N′,N′-methylene bis(acrylamide), and methacrylic acid. The Korsmeyer–Peppas model of kinetics was trailed by all nanogel formulations with non-Fickian diffusion. The results demonstrated that prepared nanogels can be employed for sustained release of the madecassoside.

## 1 Introduction

*Centella asiatica* (L.) Urb. (Apiaceae, CA) is described as a rejuvenating herb in traditional systems of medicine. The major centellosides of CA are madecassoside (MAD), asiaticoside, madecassic acid, and asiatic acid. CA depends completely on its centellosides for its therapeutic potential. MAD is a ursane-type triterpenoid saponin and acts as a neuroprotective, anti-inflammatory, antioxidant, antiapoptotic, and memory-enhancing agent. Hydrophobicity, low instability, and low bioavailability are key factors that limit the clinical use of potential drug candidates. The drawbacks of MAD include poor oral bioavailability, membrane permeability, and intestinal absorption. Hence, to address these limitations, polymeric nanocarriers are needed to sustain the release of MAD for an extended period.^1^ Kim et al. prepared *Centella asiatica*-loaded nanocrystal suspensions and reported the drug release for 4 h.^2^ Similarly, Yongsirasawad and co-workers developed *Centella asiatica* extract-loaded gelatin nanoparticles using one-step desolvation method and demonstrated drug release for almost 3.5 h.^3^ Still, much work needs to be done. Therefore, to overcome the limitations of MAD, the authors made an effort to develop polymeric nanogels of chitosan for sustained delivery of MAD. Drug release is completely dependent on the swelling of nanogels. High swelling leads to greater drug loading and drug release and vice versa.^4^ Swelling, drug loading, and release studies of the developed nanogels indicated that the release of MAD is sustained for a prolonged time.

Nanoplatforms based on nanogels have become an extremely promising drug delivery system. Nanogels prepared by physical or chemical cross-linking can load both hydrophobic and hydrophilic therapeutic agents. Due to the nanosize nature of nanogels, the stability of encapsulated drugs is not only increased but also the circulation time of drugs is prolonged. The type of reaction or cleavage of chemical bonds in the nanogel’s structure has enabled them to sustain the release of drugs in a controlled fashion for a prolonged time. Nanogels made of stimuli-sensitive polymers and monomers can show diverse responsiveness, including redox, pH, and temperature, and can assist the stimuli-sensitive drug release in the microenvironments of different disorders. Hence, to improve the therapy precision and enhance the therapeutic outcomes, changes can be made in nanogels by particular ligands to achieve active targeting and improve the drug’s accumulation at the target sites of disease.^5^

Chitosan is a natural polysaccharide polymer having good biocompatibility, mucoadhesive, and nontoxic properties. Chitosan contains amine and hydroxyl groups, which are the best sites for cross-linking and polymerization with different polymers and monomers. The combination of chitosan with monomers results in a development of high thermal polymeric networks, which not only increase their swelling capability but also enhance the mechanical strength and favorable drug release profile too.^6^ Methacrylic acid is a pH-responsive monomer and employed greatly in the development of hydrogels and their micro/nanoparticulate drug carrier systems. Good biocompatibility and ease of copolymerization are two main properties which increased the use of methacrylic acid in biomedical and pharmaceutical fields.^7^

The novelty of the developed nanogels relies on the combination of chitosan with mathacrylic acid, which not only prolongs MAD release in a controlled pattern but also prevents the rapid diffusion of MAD at acidic pH values. Similarly, the encapsulated drug was also protected from enzymatic degradation, oxidation, and hydrolysis. The prepared nanogels are subjected to various characterizations and studies. Hence, scanning electron microscopy (SEM), thermogravimetric analysis (TGA), differential scanning calorimetry (DSC), zeta sizer, Fourier transform infrared spectroscopy (FTIR), and X-ray diffraction (XRD) are the various characterizations conducted for the developed nanogels. Along with these, different studies, such as swelling, sol–gel analysis, drug loading, polymer volume, drug release, and kinetic modeling, were also accomplished for the formulated nanogels. Maximum swelling and drug release were achieved with the change in the pH of the medium, indicating the pH-responsive nature of the synthesized nanogels. Drug loading increased with higher polymer contents, but decreased with the enhancement in monomer and cross-linker contents. Similarly, increasing the amount of chitosan, N′,N′-methylene bis(acrylamide), and methacrylic acid resulted in a higher gel fraction but a lower sol fraction. Thus, we can demonstrate that developed nanogels could be applied as an alternative approach for sustained delivery of drugs.

## 2 Materials and Methods

### 2.1 Materials

Madecassoside (MAD) was obtained from Top Rhyme International Co, Ltd. Methacrylic acid (Ma) and chitosan (Cs, MW: 100,000–300,000) were purchased from TCI, Tokyo Chemical Industry Co., LTD, Japan and Acros organics (New Jersey, USA), respectively. Similarly, N′,N′-methylene bis(acrylamide) (MBA) was acquired from Alfa Aesar, Lancashire, UK and ammonium persulfate (APS) was obtained from Showa (Tokyo, Japan).

### 2.2 Preparation of Chitosan-Based Nanogels

Cs-based nanogels were fabricated by a free radical polymerization technique. A solution of Cs was prepared in 1% acetic acid and kept it on stirring. After that, the initiator APS was mixed with the Cs solution. Later, Ma was poured into the mixture of the polymer and initiator. MBA is not soluble entirely in deionized distilled water; therefore, a water/ethanol mixture was used for dissolving MBA at 50 °C. Finally, MBA was poured into the aforementioned mixture, and the mixture was stirred until the formation of a transparent solution. Nitrogen gas was purged for removing dissolved oxygen. The solution was placed in glass molds, which were positioned for 7 h at 70 °C in a water bath. The synthesized gel was passed through sieve no. 20 initially and washed by a water and ethanol mixture. The macroparticles were positioned for dryness in a vacuum oven. The dried macroparticles were passed through sieve no. 625 again to achieve fine particles of nanogels. The prepared nanogels were further characterized and studies. A formulation set of prepared nanogels is indicated in Table 1.

**Table 1:** Feed Ratio Scheme for Formulation of Chitosan-Based Nanogels

F. code	polymer Csg/20 g	monomer Mag/20 g	initiator APSg/20 g	cross-linker MBAg/20 g
*T*-1	0.2	2	0.1	3
*T*-2	0.3	2	0.1	3
*T*-3	0.4	2	0.1	3
*T*-4	0.2	2	0.1	3
*T*-5	0.2	3	0.1	3
*T*-6	0.2	4	0.1	3
*T*-7	0.2	2	0.1	3
*T*-8	0.2	2	0.1	4
*T*-9	0.2	2	0.1	5

### 2.3 Characterization

#### 2.3.1 DSC

The melting point of Cs and the developed nanogels was evaluated by DSC (PerkinElmer DSC 4000) analysis. 5 mg samples of Cs and prepared nanogels were placed in an aluminum pan. A constant nitrogen flow of 10 mL/min within a temperature range of 50–400 °C was kept throughout the scanning process. Heating rate was kept constant at 10 °C/min throughout the study.

#### 2.3.2 TGA

Thermal stability of Ca and synthesized nanogels was investigated by TGA (PerkinElmer Simultaneous Thermal Analyzer STA 8000). Thus, weighed samples (0.5–5 mg) of Cs and developed nanogel were positioned in an aluminum pan. Scanning of samples was carried out at a heating rate of 10 °C/min under a constant inert flow of nitrogen within temperature range of 40 to 600 °C.

#### 2.3.3 SEM

The surface morphology of the fabricated nanocarrier system was evaluated by SEM (JSM-5300 model, JEOL, Tokyo, Japan). The sample was sprinkled on double-adhesive tape stuck to an aluminum stub. A gold sputter module was used to coat the stub with gold under an argon atmosphere. The sample was scanned with various magnifications.^8^

#### 2.3.4 XRD

XRD (XRD-6000, Shimadzu, Tokyo, Japan) was performed to evaluate the nature of Cs and chitosan nanogels. Theta was kept constant within 10 to 60 range at a rate of 2θ/min throughout the magnification process.^8^

#### 2.3.5 FTIR

FTIR was performed to determine the structural configuration and interaction of drug formulation using attenuated total reflectance (ATR) technology (NICOLET 380 FTIR (Thermo Fisher Scientific, Ishioka, Japan)). The spectra of reagents, drug, unloaded and loaded nanogels were obtained within a scanning range of 500–4000 cm–1.^8^

### 2.4 Sol–gel Analysis

The amount of reactants converted into product was determined by a sol–gel analysis. Hence, a Soxhlet extraction technique was used for the estimation of soluble and insoluble parts of the synthesized nanogels. Hence, nanogels of precise quantity were subjected to Soxhlet extraction for almost 12 h. After that, nanogels were positioned for dehydration in the vacuum oven and weighed again.^9,10^Eqs 1 and 2 were employed for the calculation of sol–gel fraction.
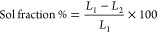
1
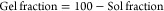
2

*L*_1_ shows the initial weight of the dried nanogels, while *L*_2_ represents the final weight after the extraction process.

### 2.5 Swelling Study

The swelling behavior of formulated nanogels was analyzed in order to determine their swelling index at pH 1.2, 4.6, and 7.4. Therefore, nanogels of specific quantity were placed in dialysis bags and then deep in the respective pH medium. Samples were recorded at predetermined intervals of time after blotting with filter paper and then returned back into the medium. This study was carried out until an equilibrium swelling was attained.^11^Eq 3 was employed for the calculation of swelling index.
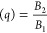
3*q* indicates the dynamic swelling, *B*_1_ represents the initial weight of dried nanogels before swelling, and *B*_2_ shows the final weight after swelling at time *t*.

### 2.6 Polymer Volume Fraction

The amount of polymer consumed during the swelling process at the same swelling pH values was determined by polymer volume (V2,s) study.^12^eq 4 was applied for the calculation of polymer volume fraction.
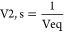
4

Veq demonstrates the equilibrium volume swelling data.

### 2.7 Drug Loading

A specific amount of prepared nanogels (500 mg) was submerged into 1% (w/v) aqueous drug solution. Sonication was performed for 30 min, and then, nanogels were placed at room temperature for 24 h. Later, nanogel’s lyophilization was performed for 24 h to remove entrapped solvent.^13,14^

### 2.8 Dissolution Study

Drug release studies were conducted for the developed nanogels at the same swelling pH values. Hence, a specific quantity of drug-loaded nanogels (200 mg) was taken in a dialysis bag and submerged in 500 mL respective buffer solution in calibrated 8 station dissolution test apparatus at body temperature with 50 rpm. Aliquots were taken at predefined time intervals, and fresh medium of equal volume was added back into the dissolution apparatus to keep the constant volume. The quantity of drug released from the prepared nanogels was quantified at λ_max_ 210 nm by using a UV-spectrophotometer (U-5100, 3J2–0014, Tokyo, Japan).^15^

### 2.9 Kinetic Modeling

The deduction of drug release from the prepared nanogels was estimated by using various kinetic models.^16^

### 2.10 Statistical Analysis

Statistical analysis for all experimental data was determined by SPSS Statistic software 22.0 (IBM Corp, Armonk, NY, USA). Student’s *t* test was applied for the difference determination between the tests and were considered significant (*p* <0.05).

## 3 Results and Discussion

### 3.1 Preparation of Chitosan-Based Nanogels

Polymeric nanogels of Cs were developed by cross-linking and polymerization process for the sustained release of MAD. Hence, Cs was polymerized with Ma by MBA in the presence of APS. Various ratios of Cs, Ma, and MBA were tried in order to check their effects on prepared nanogels. It was observed that a high concentration of Cs resulted in maximum swelling, drug loading, and release from the prepared nanogels, while Ma and MBA showed reverse effects. The main cause could be linked to the high bulk and cross-linked networks of the prepared nanogels.

### 3.2 DSC Analysis

**Figure 1:**
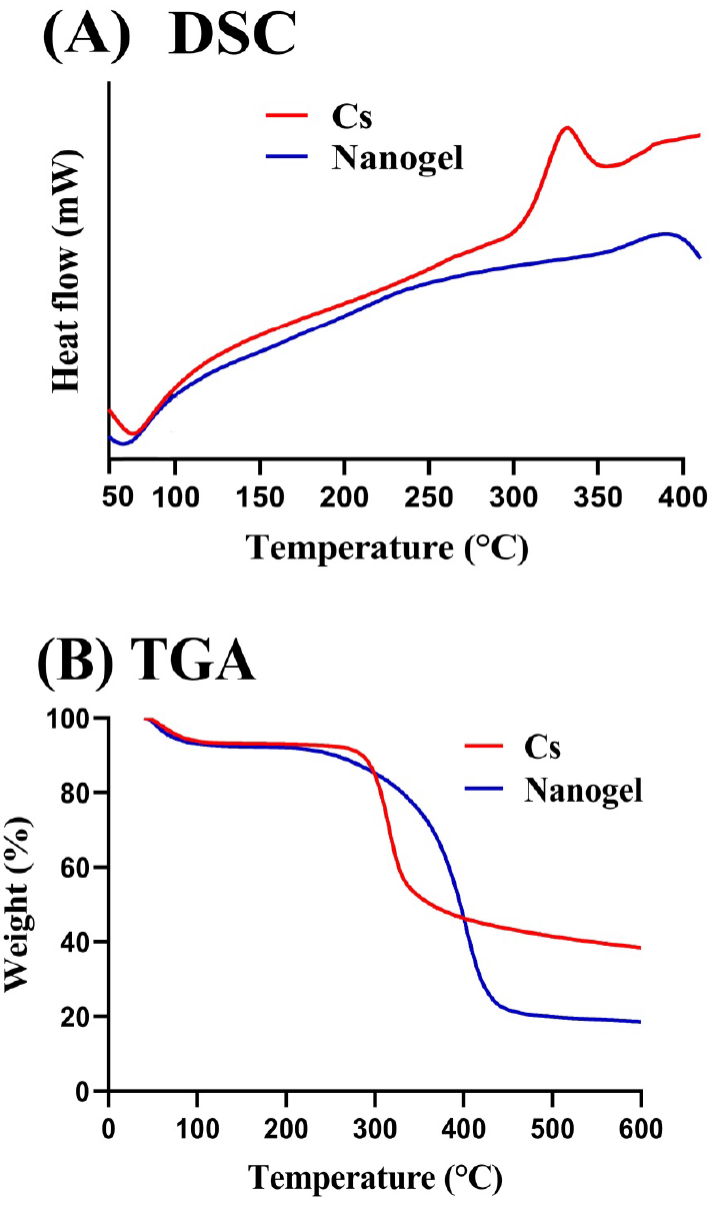
(A) DSC and (B) TGA of chitosan and chitosan-based nanogels.

DSC of Cs and prepared nanogels is indicated in Figure 1A. Decomposition was observed by the DSC of Cs within 50–20 °C temperature range with a broad endothermic peak, indicating the degradation of N-acetyl and amino groups. An exothermic peak at 340 °C was identified by Cs. Onward temperature resulted in Cs degradation. Similarly, the same endothermic peak of Cs was exhibited by the DSC of prepared nanogels with a slightly higher temperature range while the exothermic peak of the Cs was relocated from 340 to 390 °C in prepared nanogels. The endothermic peak basically indicated the glass transition temperature (*T*_g_) of the developed nanogel, which is greater than that of unreacted Cs. The enhancement in the glass transition temperature (*T*_g_) of prepared nanogel indicated strong polymerization and cross-linking of Cs with other reactive components. Due to strong cross-linking, the flexibility of the polymeric networks was decreased to undergo segmental motion. The increase in the thermal stability and high transition temperature of the prepared nanogel may be either higher intermolecular hydrogen bonding or covalent bonding.^17^ Nasir et al. synthesized polymeric gels of pluronic F-127 and reported maximum thermal stability for the synthesized gels compared to pure pluronic F-127.^18^

### 3.3 TGA

A loss in weight of Cs and prepared nanogels was determined by their TGA (Figure 1B). A 12% weight loss was detected by the TGA of Cs at 118 °C because of water evaporation. Further weight loss of 38% was detected at onward temperatures up to 308 °C. Degradation of Cs was started, and it continued until the end of the pyrolysis process. On the other hand, a very small quantity of loss in weight i.e., 4–5% was detected at 198 °C by the TGA of fabricated nanogels, related to water loss of Cs. Further reduction of 75% was seen as temperature approached 450 °C. Onward temperature resulted in nanogel’s degradation. Hence, it can be concluded from the TGA of fabricated nanogels that the thermal stability of polymer was increased because of its grafting and cross-linking with other excipients. The prepared networks of nanogel showed greater thermal stability compared to pure Cs. The high thermal stability of the polymeric nanogels specified a strong intermolecular interaction among the naogel contents, which are produced because of polymerization process.^19−21^ Shoukat et al. prepared polymeric nanogels and indicated high thermal stability for the formulated networks compared to unreacted excipients.^22^

### 3.4 SEM

**Figure 2:**
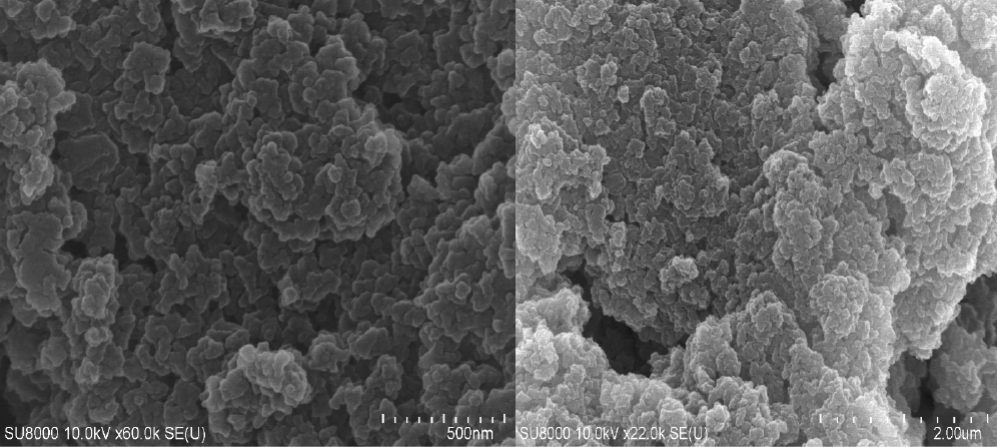
SEM of a chitosan-based nanogel.

The surface morphology of the formulated nanogels is indicated in Figure 2. A compact and high cross-linked structure of nanogels can be seen, which may be attributed to strong cross-linking of Cs with other nanogel contents. A few pores can be seen which are responsible for water penetration into the nanogel networks.^23^ Average particle size was found within the range of 100 nm, which is appropriate for maximum swelling and drug release. Particle size is influenced highly by the quantity of excipients used in the preparation of a carrier system. The surface of the nanogels will be large if the size of the particles is small and vice versa.^24^

### 3.5 XRD Analysis

**Figure 3:**
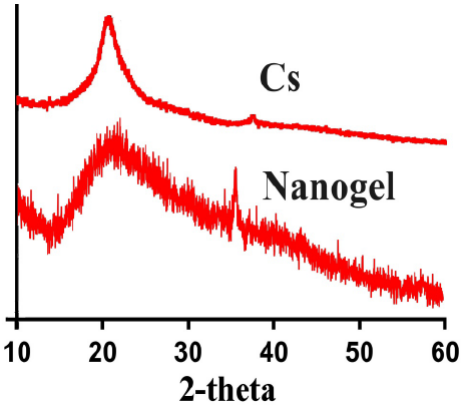
XRD of chitosan and chitosan-based nanogels.

The physical state of Cs and developed nanogels was investigated by XRD analysis (Figure 3). High intense peaks were shown by CS XRD at 2θ = 18.91° and 39.12°. Due to the polymerization of Cs with other nanogel contents, a decline in crystallinity of Cs was observed as indicated by XRD analysis of prepared nnaogels. The reduction in crystallinity of Cs indicated the successful polymerization of Cs with other components of nanogels.^25^ Abdullah and coworkers prepared polymeric hydrogels and reported a reduction in crystallinity of the reagents by prepared hydrogels.^26^ All of this indicates that the low crystallinity of prepared nanogels is mostly caused by a decrease in the crystallinity of chitosan and vice versa.

### 3.6 FTIR Analysis

**Figure 4:**
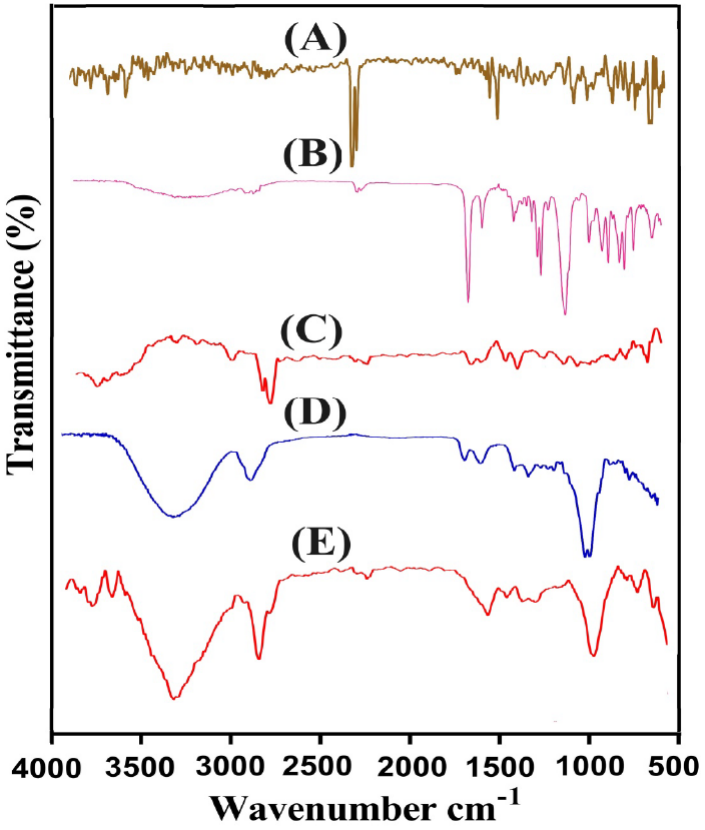
FTIR spectra of (A) of Cs, (B) Ma, (C) unloaded chitosan-based nanogels, (D) MAD, and (E) drug-loaded chitosan-based nanogels.

FTIR spectra of Cs, MA, prepared nanogels, MAD, and drug-loaded nanogels are indicated in Figure 4. FTIR spectrum of Cs (Figure 4A) revealed stretching vibrations of NH by peaks at 2858 and 3438 cm^–1^. Similarly functional groups, such as carbonyl, N–H, and C–N indicated stretching vibration by bands at 1648, 1602, and 1379 cm^–1^, respectively.^27^ FTIR spectra of Ma (Figure 4B) exhibited an asymmetric stretching vibration of C–H by a band at 2988 cm^–1^. Similarly, the stretching vibration of COOH and C=C was perceived at 1698 and 1438 cm^–1^, respectively. A change was observed in a few bands of Cs and Ma after polymerization reaction, as indicated by the FTIR spectrum of prepared nanogels (Figure 4C). The projecting bands of Cs and Ma at 1379, 1648, and 1438, and 1698 cm^–1^ were shifted to 1404, 1680, 1498, and 1712 cm^–1^ peaks of synthesized nanogels, indicating the synthesis of nanogels. Similarly, MAD (Figure 4D) exhibited its FTIR spectra by characteristic peaks at 3848 and 3750 cm^–1^ allocated to the stretching vibration of −OH, while bands at 2970 and 2872 cm^–1^ indicated stretching vibrations of C–H bonds. Likewise, C=C exhibited a stretching vibration by a peak at 1538 cm^–1^. Absorptions bands at 1218, 810, and 637 cm^–1^ indicated C–O–C and C–C stretching vibrations.^28^ Due to the encapsulation of MAD by fabricated nanogels, the intensity of certain peaks of MAD, i.e., 1538 and 2970 cm^–1^ were decreased to 1520 and 2950 cm^–1^ bands of the formulated nanogels (Figure 4E), respectively. There was no interaction seen between the MAD and nanogel’s excipients.^29^

### 3.7 Sol–gel Analysis

The soluble and insoluble parts of the nanogels were determined by the sol–gel fraction. The gelation among nanogels was enhanced with the high composition of Cs and Ma (Table 2). Free radicals were produced in greater quantities with the high integration of polymer and monomer, resulting in greater gelation among nanogel contents, and so greater gelation was observed. Likewise, cross-linking density increased with the high concentration of MBA because cross-linking among nanogel contents is completely dependent on MBA. Therefore, a rise in gelation was detected as the concentration of MBA enhanced (Table 2). On the other hand, a reduction was detected in the soluble sol fraction of the nanogels with the high integration of Cs, Ma, and MBA because sol and gel fractions are inversely proportional to each other and vice versa. Khalid and coworkers developed polymeric hydrogels and reported high gel while low sol fractions with the high integration of hydrogel contents.^30^

**Table 2:** Sol-Gel and Drug Loading of Chitosan-Based Nanogels

F. code	sol-fraction (%)	gel fraction (%)	drug loaded (mg)/500 mg of dry gels extraction method
*T*-1	13.47	86.53	148.32 ± 0.80
*T*-2	11.61	88.39	157.10 ± 0.72
*T*-3	10.12	89.88	172.52 ± 1.12
*T*-4	13.47	86.53	148.32 ± 0.80
*T*-5	12.13	87.87	135.40 ± 0.52
*T*-6	11.02	88.98	128.12 ± 1.20
*T*-7	13.47	86.53	148.32 ± 0.80
*T*-8	10.08	89.92	141.45 ± 0.93
*T*-9	8.92	91.08	137.82 ± 0.64

### 3.8 Swelling Study

**Figure 5:**
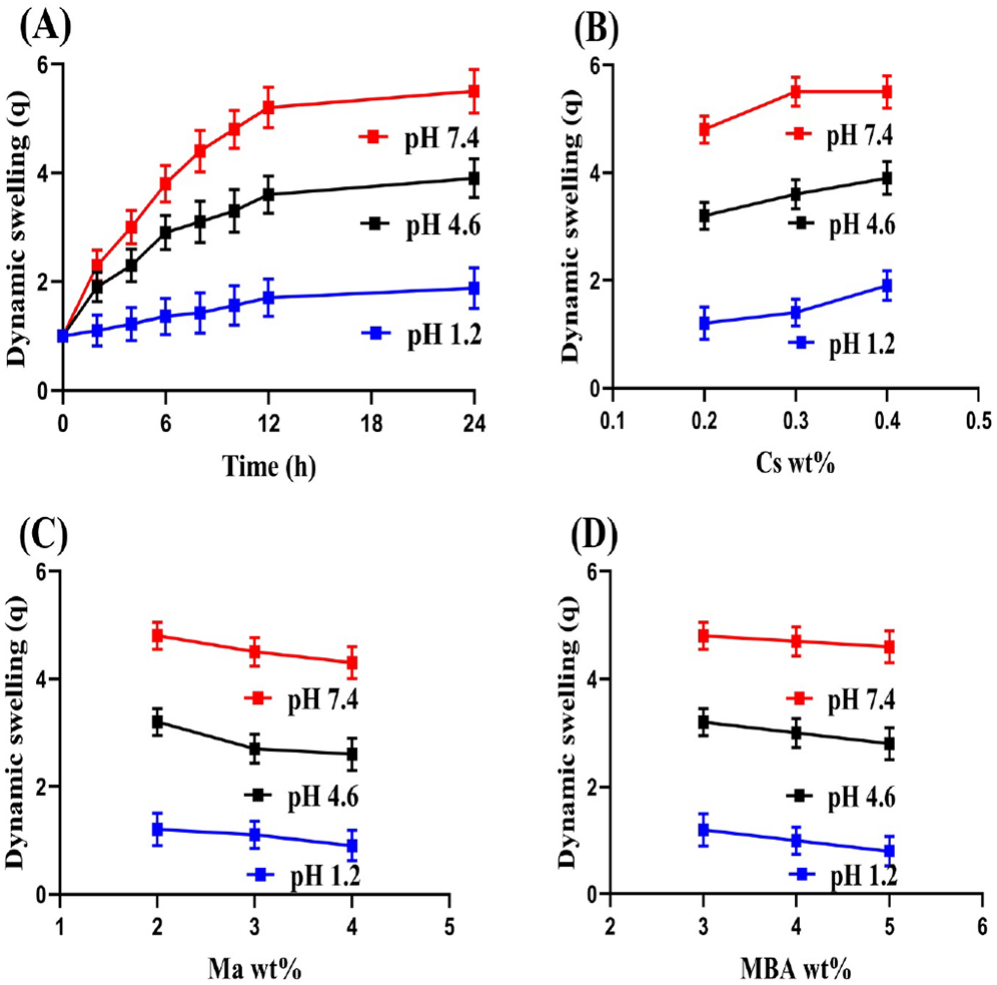
Effect of (A) pH, (B) Cs, (C) Ma, and (D) MBA on swelling of chitosan-based nanogels.

The pH-sensitive nature of the developed nanogels was evaluated at pH 1.2, 4.6, and 7.4 by swelling studies. A low swelling index was seen at pH 1.2 compared to pH 4.6 and 7.4 as illustrated in Figure 5A. Cs contains the NH_2_ group, which led to protonation at pH 1.2. Furthermore, a reduction in the NH_2_ group was observed due to the grafting of Cs with other nanogel contents and, thus, low swelling was detected at low pH 1.2.^31^ Similarly, Ma contains OH and COOH functional groups, which leads to protonation at low pH 1.2. A conjugate was formed through hydrogel bonding by the functional groups of Cs and MA with counterions, resulting in reduced charge density of the same functional groups and low swelling of fabricated nanogels at pH 1.2. On the other hand, a change in the swelling index was seen with the pH change of the medium. Increased swelling, particularly at high pH 7.4, was exhibited by the prepared nanogels. The reason can be linked with the deprotonation of Cs and Ma functional groups, which leads to a high charge density of the same functional groups. Therefore, due to strong electrostatic repulsive forces, a rise was detected in nanogel’s swelling at pH 4.6 and 7.4.^32−34^

The swelling index of the formulated nanogels was also influenced by the integration of different ratios of Cs, MBA, and Ma (Figure 5B–D). High concentration of Cs resulted in the generation of high NH_2_ groups, which led to high swelling of nanogels. The hydrophilicity of the polymeric nanogels is increased with the high integration of Cs, hence more swelling was observed.^35^ Contrary to Cs, swelling index of nanogels was reduced with high composition of Ma and MBA. The motility and flexibility of the nanogels were decreased due to the strong cross-linking of Ma and MBA with polymer and initiator, and as a result, low swelling was perceived with the high integration of MBA and Ma.^36−38^

### 3.9 Polymer Volume Fraction

Polymer volume study was carried out with the purpose of determining the amount of nanogel contents consumed during the swelling process at pH 1.2, 4.6, and 7.4, as shown in Table 3. The different concentrations of Cs, Ma, and MBA have influenced the polymer volume fraction as swelling but in an inverse way. Low polymer volume values were achieved with the higher concentration of Cs. On the other hand, higher polymer volume fractions was seen with the high concentration of Ma and MBA. The decrease in polymer volume fraction of Cs while increasing MA and MBA may be related to the high and low swelling degree of the formulated nanogels at different pH values. The high polymer volume fraction at pH 1.2 and 4.6, while low at pH 7.4, demonstrated strong swelling capability of prepared nanogels at pH 7.4 in the order of pH 7.4 > 4.6 > 1.2. Thus, we can conclude that a high polymer volume fraction of the prepared nanogels is achieved with low swelling index because of the inverse relationship between the swelling and polymer volume fraction.^12^

**Table 3:** Polymer Volume Fraction of Chitosan-Based Nanogels

	Polymer volume fraction		
Formulation code	pH 1.2	pH 4.6	pH 7.4
*T*-1	0.833	0.312	0.204
*T*-2	0.714	0.277	0.192
*T*-3	0.590	0.256	0.181
*T*-4	0.833	0.312	0.204
*T*-5	0.901	0.370	0.222
*T*-6	0.950	0.384	0.232
*T*-7	0.833	0.312	0.204
*T*-8	0.870	0.333	0.212
*T*-9	0.930	0.357	0.217

### 3.10 Drug Loading and Drug Release Studies

**Figure 6:**
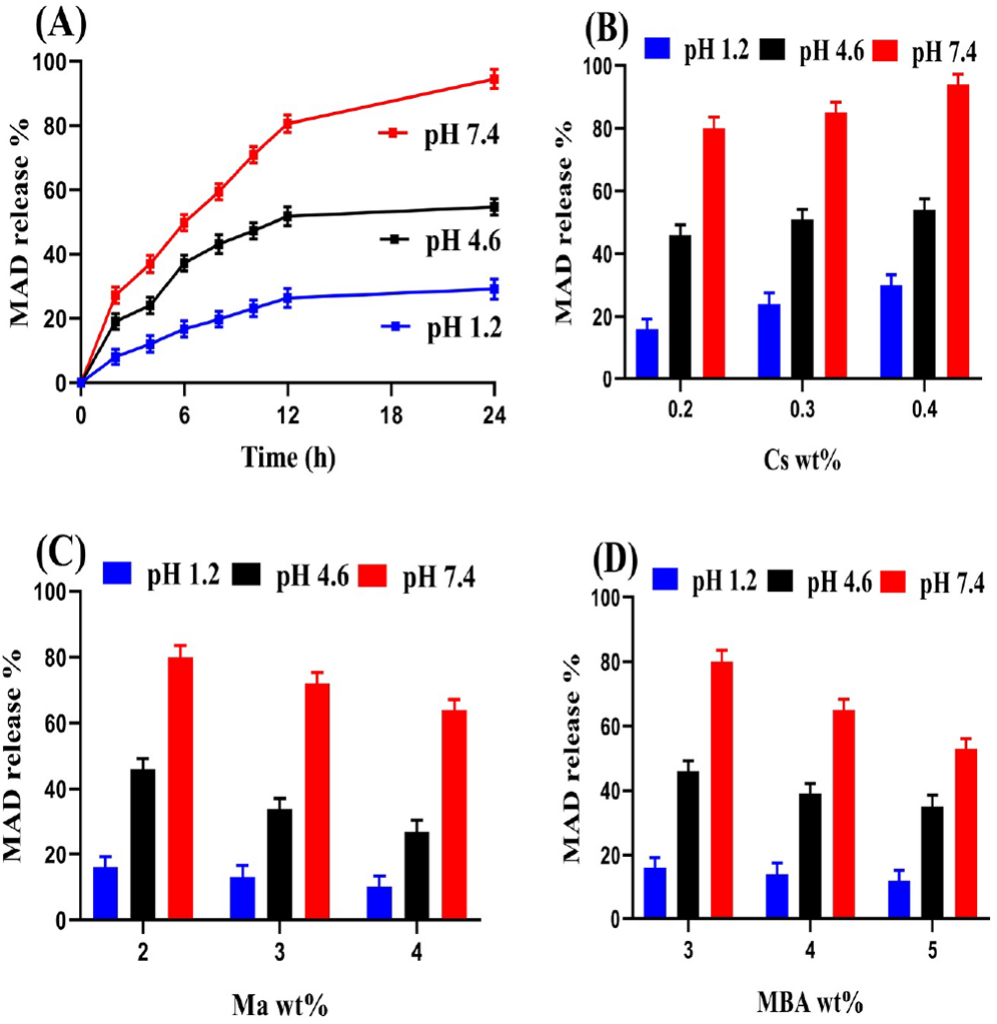
Effect of (A) pH, (B) Cs, (C) Ma, and (D) MBA on release of drug from chitosan-based nanogels.

The pH-responsive nature of the prepared nanogels was also evaluated by a dissolution test at the same swelling pH values. A low drug release was seen at pH 1.2 as compared to pH 4.6 and 7.4 (Figure 6A). The possible reason was the protonation of the COOH, OH, and NH_2_ groups of Ma and Cs, which led to almost low release of the drug at pH 1.2. On the other hand, high release of the drug was perceived at pH 4.6 and 7.4. Due to OH and COOH group’s deprotonation, increase in charge density of prepared polymeric nanogels was observed. As a result, strong electrostatic repulsive forces were formed, which led to high drug release especially at pH 7.4.^39^

Both drug loading and release were influenced by the high integration of Cs, Ma, and MBA as illustrated in Table 2 and Figure 6B–D. Greater drug loading and release was achieved with the high Cs concentration because a large amount of Cs indicated a high availability of NH_2_ groups, and as a result, maximum drug loading and release was achieved.^40−42^ Unlike Cs, drug loading as well as drug release were decreased with high integration of Ma and MBA. The main reason is the development of a very hard network of nanogels, which reduced the motility and flexibility of the developed nanogels and as a result, low drug loading and release was achieved. The pore size of the prepared nanogels was reduced, due to which sufficient amount of water was not penetrated into the nanogel networks, resulting in low swelling and vice versa.^43^

### 3.11 Kinetic Modeling

Water molecules are diffused into the fabricated nanogels due to the osmotic pressure gradient when nanogels are placed in water. Due to diffusion of water, nanogels swell, resulting in the channel opening, and thus drug is released alternatively. Regression coefficient “*r*” values close to 1 indicated the most suitable fit model of kinetics. “*r*” values of all formulations have been shown in Table 4. All formulations of developed nanogels tracked the Korsmeyer–Peppas model due to their “*r*” values, which are higher than the rest of the kinetic models. Moreover, “*n*” values of all formulations following the Korsmeyer–Peppas model were found within the range of 0.4513 to 0.5766, indicating non-Fickian diffusion.^44^

**Table 4:** Kinetic Modeling Release of MAD from Chitosan-Based Nanogels

				Korsmeyer–Peppas	
F. code	zero order *r*^2^	first order *r*^2^	Higuchi *r*^2^	*r*^2^	*n*
*T*-1	0.9552	0.9958	0.7092	0.9972	0.5594
*T*-2	0.9749	0.8981	0.9624	0.9860	0.5264
*T*-3	0.9733	0.8834	0.9927	0.9946	0.5236
*T*-4	0.9552	0.9958	0.7092	0.9972	0.5594
*T*-5	0.9810	0.9707	0.9107	0.9867	0.5358
*T*-6	0.9623	0.9451	0.9568	0.9873	0.5766
*T*-7	0.9552	0.9958	0.7092	0.9972	0.5594
*T*-8	0.9588	0.8334	0.9890	0.9903	0.4513
*T*-9	0.9591	0.8415	0.9966	0.9974	0.4789

## 4 Conclusion

A free radical polymerization approach was applied for the engineering of chitosan-based nanogels. TGA and DSC indicated an enhancement in the thermal stability of polymer after the polymerization and grafting process, while a rigid and hard structure was shown by SEM analysis. The decrease in chitosan’s crystallinity and preparation of nanogels were confirmed by XRD and FTIR analyses. Similarly, excellent pH-sensitivity was exhibited by synthesized nanogels as maximum drug release and swelling index were achieved at pH 7.4. An increase in the gel fraction was perceived with the high integration of Cs, Ma, and MBA, where a reduction was detected in the sol fraction. Swelling and drug release studies indicated that the release of MAD was completely dependent on the composition of Cs, Ma, and MBA and pH of the medium. Increased swelling and release of the drug were achieved with the high integration of Cs, whereas Ma and MBA revealed reverse effects. Similarly, the same effects of polymer, cross-linker, and monomer were shown on drug loading too. More than 95% drug release was observed at pH 7.4, while almost 30% and 50% drug release were seen at pH 1.2 and 4.6 within 24 h, respectively, indicating the pH-sensitive nature of the developed nanogels. “*r*″ values of the Korsmeyer–Peppas model were found to be greater than the respective kinetic models, indicating that all formulations of the prepared nanogels followed this model. Similarly, “*n*” values indicated non-Fickian diffusion. A swelling study with higher polymer volume fraction at pH 1.2 and 4.6 compared to pH 7.4 indicated pH dependency and increased swelling degree of the formulated nanogels in a high pH medium. According to release kinetic models, all nanogel formulations followed the Korsmeyer–Peppas model of kinetics because their “*r*” values were very close to 1 when compared to the other kinetic models. Hence, it can be concluded from the results that chitosan-based nanogels have the potential to be employed as pH-responsive carriers for sustained drug delivery. Further, toxicity tests and pharmacokinetic studies are being conducted to confirm the effectiveness of the prepared formulations in the future.

## References

[ref1] Kunjumon R.; Viswanathan G.; Baby S. Biocompatible madecassoside encapsulated alginate chitosan nanoparticles, their anti-proliferative activity on C6 glioma cells. Carbohydr. Polym. Technol. Appl. 2021, 2, 100106 10.1016/j.carpta.2021.100106.

[ref2] Kim E.; Park J. S.; Kim M. S.; Jeong M. Y.; Park H. J.; Choi J. H.; Seo J. H.; Choi Y. S.; Kang M. J. High-Payload Nanosuspension of Extract for Improved Skin Delivery with No Irritation. Int. J. Nanomed. 2021, 16, 7417–7432. 10.2147/IJN.S335039. PMC8573141 34764648

[ref3] Yongsirasawad K.; Yasurin P.; Asavasanti S.; Amornraksa S.; Sriariyanun M. The Drug Delivery System of Centella asiatica extract-loaded Gelatin Nanoparticles using of One-step desolvation Method. In The 2018 8th International Conference, Association for Computing Machinery, 2018.

[ref4] Suhail M.; Fang C. W.; Chiu I. H.; Khan A.; Wu Y. C.; Lin I. L.; Tsai M. J.; Wu P. C. Synthesis and Evaluation of Alginate-Based Nanogels as Sustained Drug Carriers for Caffeine. ACS Omega 2023, 8 (26), 23991–24002. 10.1021/acsomega.3c02699. 37426260 PMC10324385

[ref5] Yin Y.; Hu B.; Yuan X.; Cai L.; Gao H.; Yang Q. Nanogel: A Versatile Nano-Delivery System for Biomedical Applications. Pharmaceutics 2020, 12, 290 10.3390/pharmaceutics12030290. 32210184 PMC7151186

[ref6] Qu B.; Luo Y. Chitosan-based hydrogel beads: Preparations, modifications and applications in food and agriculture sectors–A review. Int. J. Biol. Macromol. 2020, 152, 437–448. 10.1016/j.ijbiomac.2020.02.240. 32097742

[ref7] Milosavljević N. B.; Milašinović N. Z.; Popović I. G.; Filipović J. M.; Kalagasidis Krušić M. T. Preparation and characterization of pH-sensitive hydrogels based on chitosan, itaconic acid and methacrylic acid. Polym. Int. 2011, 60 (3), 443–452. 10.1002/pi.2967.

[ref8] Suhail M.; Fang C.-W.; Chiu I.-H.; Ullah H.; Khan A.; Tsai M.-J.; Wu P.-C. Preparation of chondroitin sulfate and polyvinyl alcohol hydrogels as drug carriers. Appl. Surf. Sci. Adv. 2023, 18, 100478 10.1016/j.apsadv.2023.100478.

[ref9] Suhail M.; Khan A.; Rosenholm J. M.; Minhas M. U.; Wu P. C. Fabrication and Characterization of Diclofenac Sodium Loaded Hydrogels of Sodium Alginate as Sustained Release Carrier. Gels 2021, 7 (1), 10 10.3390/gels7010010. 33514036 PMC7930945

[ref10] Ullah K.; Khan S. A.; Murtaza G.; Sohail M.; Manan A.; Manan A.; Afzal A. Gelatin-based hydrogels as potential biomaterials for colonic delivery of oxaliplatin. Int. J. Pharm. 2019, 556, 236–245. 10.1016/j.ijpharm.2018.12.020. 30553956

[ref11] Suhail M.; Wu P. C.; Minhas M. U. Using Carbomer-Based Hydrogels for Control the Release Rate of Diclofenac Sodium: Preparation and In Vitro Evaluation. Pharmaceuticals 2020, 13 (11), 399 10.3390/ph13110399. 33212866 PMC7698439

[ref12] Badshah S. F.; Akhtar N.; Minhas M. U.; Khan K. U.; Khan S.; Abdullah O.; Naeem A. Porous and highly responsive cross-linked β-cyclodextrin based nanomatrices for improvement in drug dissolution and absorption. Life Sci. 2021, 267, 118931 10.1016/j.lfs.2020.118931. 33359243

[ref13] Suhail M.; Wu P. C.; Minhas M. U. Development and characterization of pH-sensitive chondroitin sulfate-co-poly(acrylic acid) hydrogels for controlled release of diclofenac sodium. J. Saudi Chem. Soc. 2021, 25 (4), 101212 10.1016/j.jscs.2021.101212.

[ref14] Khan S.; Ranjha N. M. Effect of degree of cross-linking on swelling and on drug release of low viscous chitosan/poly (vinyl alcohol) hydrogels. Polym. Bull. 2014, 71 (8), 2133–2158. 10.1007/s00289-014-1178-2.

[ref15] Khan K. U.; Minhas M. U.; Sohail M.; Badshah S. F.; Abdullah O.; Khan S.; Munir A.; Suhail M. Synthesis of PEG-4000-co-poly (AMPS) nanogels by cross-linking polymerization as highly responsive networks for enhancement in meloxicam solubility. Drug Dev. Ind. Pharm. 2021, 47 (3), 465–476. 10.1080/03639045.2021.1892738. 33651645

[ref16] Peppas N. A.; Sahlin J. J. A simple equation for the description of solute release. III. Coupling of diffusion and relaxation. Int. J. Pharm. 1989, 57 (2), 169–172. 10.1016/0378-5173(89)90306-2.

[ref17] Shah S. A.; Sohail M.; Karperien M.; Johnbosco C.; Mahmood A.; Kousar M. Chitosan and carboxymethyl cellulose-based 3D multifunctional bioactive hydrogels loaded with nano-curcumin for synergistic diabetic wound repair. Int. J. Biol. Macromol. 2023, 227, 1203–1220. 10.1016/j.ijbiomac.2022.11.307. 36473525

[ref18] Nasir N.; Ahmad M.; Minhas M. U.; Barkat K.; Khalid M. F. pH-responsive smart gels of block copolymer [pluronic F127-co-poly (acrylic acid)] for controlled delivery of Ivabradine hydrochloride: Its toxicological evaluation. J. Polym. Res. 2019, 26, 1–15. 10.1007/s10965-019-1872-8.

[ref19] Wei W.; Hu X.; Qi X.; Yu H.; Liu Y.; Li J.; Zhang J.; Dong W. A novel thermo-responsive hydrogel based on salecan and poly (N-isopropylacrylamide): Synthesis and characterization. Colloids Surf., B 2015, 125, 1–11. 10.1016/j.colsurfb.2014.10.057. 25460596

[ref20] Hu X.; Feng L.; Wei W.; Xie A.; Wang S.; Zhang J.; Dong W. Synthesis and characterization of a novel semi-IPN hydrogel based on Salecan and poly (N, N-dimethylacrylamide-co-2-hydroxyethyl methacrylate). Carbohydr. Polym. 2014, 105, 135–144. 10.1016/j.carbpol.2014.01.051. 24708962

[ref21] Ray M.; Pal K.; Anis A.; Banthia A. Development and characterization of chitosan-based polymeric hydrogel membranes. Des. Monomers Polym. 2010, 13 (3), 193–206. 10.1163/138577210X12634696333479.

[ref22] Shoukat H.; Pervaiz F.; Rehman S.; Akram F.; Noreen S.; Khan K. U.; Basit A.; Ashraf M. A. Development, in vitro and in vivo evaluation of β-cyclodextrin/Polyvinyl alcohol-co-poly (2-acrylamide-2-methylpropane sulfonic acid) cross-linked hybrid IPN-nanogels to improve the dissolution and absorption of anti-hyperlipidemic drug. Polym.-Plast. Technol. Mater. 2023, 62 (14), 1945–1967. 10.1080/25740881.2023.2237130.

[ref23] Sinha P.; Ubaidulla U.; Nayak A. K. Okra (Hibiscus esculentus) gum-alginate blend mucoadhesive beads for controlled glibenclamide release. Int. J. Biol. Macromol. 2015, 72, 1069–1075. 10.1016/j.ijbiomac.2014.10.002. 25312603

[ref24] Mahmood M. A.; Madni A.; Rehman M.; Rahim M. A.; Jabar A. Ionically Cross-Linked Chitosan Nanoparticles for Sustained Delivery of Docetaxel: Fabrication, Post-Formulation and Acute Oral Toxicity Evaluation. Int. J. Nanomed. 2019, 14, 10035–10046. 10.2147/IJN.S232350. PMC6929931 31908458

[ref25] Hu X.; Wang Y.; Zhang L.; Xu M.; Dong W.; Zhang J. Redox/pH dual stimuli-responsive degradable Salecan-g-SS-poly (IA-co-HEMA) hydrogel for release of doxorubicin. Carbohydr. Polym. 2017, 155, 242–251. 10.1016/j.carbpol.2016.08.077. 27702509

[ref26] Abdullah O.; Usman Minhas M.; Ahmad M.; Ahmad S.; Barkat K.; Ahmad A. Synthesis, optimization, and evaluation of polyvinyl alcohol-based hydrogels as controlled combinatorial drug delivery system for colon cancer. Adv. Polym. Technol. 2018, 37 (8), 3348–3363. 10.1002/adv.22119.

[ref27] Ahmad S.; Minhas M. U.; Ahmad M.; Sohail M.; Abdullah O.; Badshah S. F. Preparation and evaluation of skin wound healing chitosan-based hydrogel membranes. AAPS PharmScitech 2018, 19 (7), 3199–3209. 10.1208/s12249-018-1131-z. 30171450

[ref28] Thomas M. T.; Kurup R.; Johnson A. J.; Chandrika S. P.; Mathew P. J.; Dan M.; Baby S. Elite genotypes/chemotypes, with high contents of madecassoside and asiaticoside, from sixty accessions of Centella asiatica of south India and the Andaman Islands: For cultivation and utility in cosmetic and herbal drug applications. Ind. Crops Prod. 2010, 32 (3), 545–550. 10.1016/j.indcrop.2010.07.003.

[ref29] Ahmad W.; Khalid I.; Barkat K.; Minhas M. U.; Khan I. U.; Syed H. K.; Mali N. S.; Jamshed A.; Ikram A.; Badshah M. Development and evaluation of polymeric nanogels to enhance solubility of letrozole. Polym. Bull. 2023, 80 (4), 4085–4116. 10.1007/s00289-022-04248-5.

[ref30] Khalid I.; Ahmad M.; Minhas M. U.; Barkat K. Synthesis and evaluation of chondroitin sulfate based hydrogels of loxoprofen with adjustable properties as controlled release carriers. Carbohydr. Polym. 2018, 181, 1169–1179. 10.1016/j.carbpol.2017.10.092. 29253946

[ref31] Malik N. S.; Ahmad M.; Minhas M. U.; Tulain R.; Barkat K.; Khalid I.; Khalid Q. Chitosan/xanthan gum based hydrogels as potential carrier for an antiviral drug: Fabrication, characterization, and safety evaluation. Front. Chem. 2020, 8, 50 10.3389/fchem.2020.00050. 32117876 PMC7010646

[ref32] Hussain A.; Khalid S. H.; Qadir M. I.; Massud A.; Ali M.; Khan I. U.; Saleem M.; Iqbal M. S.; Asghar S.; Gul H. Water uptake and drug release behaviour of methyl methacrylate-co-itaconic acid [P(MMA/IA)] hydrogels cross-linked with methylene bis-acrylamide. J. Drug Delivery Sci. Technol. 2011, 21 (3), 249–255. 10.1016/S1773-2247(11)50034-6.

[ref33] Bukhari S. M. H.; Khan S.; Rehanullah M.; Ranjha N. M. Synthesis and characterization of chemically cross-linked acrylic acid/gelatin hydrogels: Effect of pH and composition on swelling and drug release. Int. J. Polym. Sci. 2015, 2015, 187961 10.1155/2015/187961.

[ref34] Lim S. L.; Tang W. N. H.; Ooi C. W.; Chan E. S.; Tey B. T. Rapid swelling and deswelling of semi-interpenetrating network poly (acrylic acid)/poly (aspartic acid) hydrogels prepared by freezing polymerization. J. Appl. Polym. Sci. 2016, 133 (24), 43515 10.1002/app.43515.

[ref35] Mandal B.; Ray S. K. Swelling, diffusion, network parameters and adsorption properties of IPN hydrogel of chitosan and acrylic copolymer. Mater. Sci. Eng.: C 2014, 44, 132–143. 10.1016/j.msec.2014.08.021. 25280689

[ref36] Khalid S.; Qadir M.; Massud A.; Ali M.; Rasool M. Effect of degree of cross-linking on swelling and drug release behaviour of poly (methyl methacrylate-co-itaconic acid)[P (MMA/IA)] hydrogels for site specific drug delivery. J. Drug Delivery Sci. Technol. 2009, 19 (6), 413–418. 10.1016/S1773-2247(09)50085-8.

[ref37] Peng G.; Xu S.; Peng Y.; Wang J.; Zheng L. A new amphoteric superabsorbent hydrogel based on sodium starch sulfate. Bioresour. Technol. 2008, 99 (2), 444–447. 10.1016/j.biortech.2007.01.018. 17360178

[ref38] Wu W.; Wang D.-S. A fast pH-responsive IPN hydrogel: Synthesis and controlled drug delivery. React. Funct. Polym. 2010, 70 (9), 684–691. 10.1016/j.reactfunctpolym.2010.06.002.

[ref39] Bera R.; Dey A.; Chakrabarty D. Synthesis, Characterization, and drug release study of acrylamide- co -itaconic acid based smart hydrogel. Polym. Eng. Sci. 2015, 55 (1), 113–122. 10.1002/pen.23874.

[ref40] Khalid I.; Ahmad M.; Usman Minhas M.; Barkat K.; Sohail M. Cross-Linked Sodium Alginate-g-Poly (Acrylic Acid) Structure: A Potential Hydrogel Network for Controlled Delivery of Loxoprofen Sodium. Adv. Polym. Technol. 2018, 37 (4), 985–995. 10.1002/adv.21747.

[ref41] Agnihotri S. A.; Aminabhavi T. M. Novel interpenetrating network chitosan-poly (ethylene oxide-g-acrylamide) hydrogel microspheres for the controlled release of capecitabine. Int. J. Pharm. 2006, 324 (2), 103–115. 10.1016/j.ijpharm.2006.05.061. 16824710

[ref42] Khalid Q.; Ahmad M.; Usman Minhas M. Hydroxypropyl-β-cyclodextrin hybrid nanogels as nano-drug delivery carriers to enhance the solubility of dexibuprofen: Characterization, in vitro release, and acute oral toxicity studies. Adv. Polym. Technol. 2018, 37 (6), 2171–2185. 10.1002/adv.21876.

[ref43] Ali L.; Ahmad M.; Usman M.; Yousuf M. Controlled release of highly water-soluble antidepressant from hybrid copolymer poly vinyl alcohol hydrogels. Polym. Bull. 2014, 71 (1), 31–46. 10.1007/s00289-013-1043-8.

[ref44] Maziad N. A.; El-Hamouly S.; Zied E.; Kelani E. L.; A T.; Nasef N. R. Radiation preparation of smart hydrogel has antimicrobial properties for controlled release of ciprofloxacin in drug delivery systems. Asian J. Pharm. Clin. Res. 2015, 8 (3), 193–200.

